# The Association between Metabolic Syndrome, Bone Mineral Density, Hip Bone Geometry and Fracture Risk: The Rotterdam Study

**DOI:** 10.1371/journal.pone.0129116

**Published:** 2015-06-12

**Authors:** Taulant Muka, Katerina Trajanoska, Jessica C. Kiefte-de Jong, Ling Oei, André G Uitterlinden, Albert Hofman, Abbas Dehghan, M. Carola Zillikens, Oscar H. Franco, Fernando Rivadeneira

**Affiliations:** 1 Department of Epidemiology, Erasmus Medical Center, Rotterdam, the Netherlands; 2 Department of Internal Medicine, Erasmus Medical Center, Rotterdam, the Netherlands; 3 Netherlands Consortium for Healthy Ageing, Netherlands Genomics Inititiative, The Hague, the Netherlands; 4 Department of Internal Medicine, IJsselland Hospital, Capelle aan den Ijssel, the Netherlands; Oklahoma State University, UNITED STATES

## Abstract

The association between metabolic syndrome (MS) and bone health remains unclear. We aimed to study the association between MS and hip bone geometry (HBG), femoral neck bone mineral density (FN-BMD), and the risk of osteoporosis and incident fractures. Data of 2040 women and 1510 men participants in the third visit (1997–1999) of the Rotterdam Study (RSI-3), a prospective population based cohort, were available (mean follow-up 6.7 years). MS was defined according to the recent harmonized definition. HBG parameters were measured at the third round visit whereas FN-BMD was assessed at the third round and 5 years later. Incident fractures were identified from medical registry data. After correcting for age, body mass index (BMI), lifestyle factors and medication use, individuals with MS had lower bone width (β = -0.054, *P* = 0.003), lower cortical buckling ratio (β = -0.81, *P* = 0.003) and lower odds of having osteoporosis (odds ratio =0.56, *P* = 0.007) in women but not in men. Similarly, MS was associated with higher FN-BMD only in women (β = 0.028, *P*=0.001). In the analyses of MS components, the glucose component (unrelated to diabetes status) was positively associated with FN-BMD in both genders (β = 0.016, *P* = 0.01 for women and β = 0.022, *P* = 0.004 for men). In men, waist circumference was inversely associated with FN-BMD (β = -0.03, *P* = 0.004). No association was observed with fracture risk in either sex. In conclusion, women with MS had higher FN-BMD independent of BMI. The glucose component of MS was associated with high FN-BMD in both genders, highlighting the need to preserve glycemic control to prevent skeletal complications.

## Introduction

Osteoporosis, a systemic skeletal disease, is an important public health problem due to its increased risk for fractures, high morbidity and mortality and significant health care costs [1,2]. Different factors have been associated with osteoporosis including abdominal obesity, hypertension, dyslipidemia and abnormal glucose metabolism, which are considered components of metabolic syndrome. These components might affect bone differently. Obesity may lead to increased bone mineral density (BMD) because it is associated with higher 17β-estradiol levels and higher mechanical load, which may protect bone [3,4]. Visceral fat accumulation is associated with higher levels of pro-inflammatory cytokines, which may up-regulate receptor activators of nuclear kappa B ligand, leading to increased bone resorption and therefore decreased BMD[5–7]. Also, although hyperglycemia is a predictor of osteoporotic fractures [8], the association between high glucose levels or insulin resistance with BMD is not well defined. For example, diabetic individuals have higher BMD but increased risk of fractures [9–11]. Similarly, the evidence for associations between dyslipidemia and hypertension with bone metabolism is still inconclusive and the overall association between MS and bone health remains unclear [12–15]. Two recent meta-analyses on the association between MS and BMD and fractures, were not conclusive and whether metabolic syndrome might be associated with bone health beyond the contribution of the individual components remains unclear [16,17]. Moreover, sex-differences have been suggested in this relationship, mainly due to differences in body fat distribution between men and women, but yet not clearly defined [18–20]. Recently, a new cluster of criteria for diagnosis of MS had been presented with an emphasis on gender and ethnic differences in the measure of central obesity [21], which has not been adequately studied in relation to bone.

We aimed to study the association between MS, femoral neck BMD (FN-BMD), hip bone geometry (HBG), osteoporosis, and fractures among elderly Dutch men and women, participants of the Rotterdam Study and if these associations were independent of body mass index (BMI). We examined whether MS was associated with FN-BMD and HBG parameters in females and males using a cross-sectional design and to determine whether MS predicts FN-BMD, and incident fractures using a longitudinal design.

## Materials and Methods

### Study Population

The study was performed within the framework of the Rotterdam Study, a population-based cohort among persons ≥55 years and older living in the Ommoord district of Rotterdam, the Netherlands. The rationale and design of the Rotterdam Study is described elsewhere [22]. The study was approved by the Medical Ethical Committee of Erasmus University, and all participants gave informed consent. The present study used data from the baseline examination of the third wave of the first cohort (RS-I-3) (1997–1999), in which 2,463 females and 1750 males participated and FN-BMD was measured at the fourth round (RSI-4) (2002–2004).

### Population for Analysis

#### Metabolic syndrome, FN-BMD, hip bone geometry and osteoporosis

Among 2463 females and 1750 males, 402 women and 231 men did not have fasting samples or measures of at least on component of MS, and were therefore excluded from the analysis. Furthermore, FN-BMD at RSI-3 (1997–1999), was not assessed in 534 females and 353 males, leaving 1527 females and 1166 males for the cross-sectional analysis regarding MS, FN-BMD and osteoporosis. For the analysis concerning MS and HBG, 124 female and 48 males were further excluded because of no-available information on HBG data (S1 and S2 Figs).

#### Metabolic syndrome and fractures risk

Among 2061 females and 1519 males with information available on MS components at RSI-3, 29 subjects (21 females and 9 males) were excluded because there was no data on fracture follow-up, hence leaving 2040 females and 1510 males for the analysis on MS and fracture risk. Furthermore, for each type of fracture, prevalent cases were excluded from the analysis (S1 and S2 Figs).

### Metabolic Syndrome Definition

MS was defined according to the new criteria announced by a joint scientific statement from the International Diabetes Federation (IDF), the American Heart Association/National Heart, Lung, and Blood Institute (AHA/NHLBI), World Heart Federation, International Atherosclerosis Society and International Association for the Study of Obesity [21]. Participants with three or more of the following components were classified as having MS: (1) abdominal obesity (waist circumference (WC) ≥102 cm for men or ≥88 cm for women); (2) high triglycerides (TG) (≥150 mg/dL) (3) low HDL (≤40 mg/dL for men or ≤50 mg/dL for women) (4) elevated blood pressure (BP) (systolic BP ≥130 and/or diastolic BP ≤85 mmHg) or (5) high glucose (fasting glucose level ≥100 mg/dL). The criteria for abdominal obesity were adopted from the cutoffs for European people region [21].

### Skeletal Assessments

All events, including incident fractures and death, were reported by general practitioners (GPs) in the research area by means of a computerized system. All reported events were verified by two trained research physicians, who independently reviewed and coded the information. Subsequently, all coded events were reviewed by a medical expert for final classification. Subjects were followed from their baseline visit until January 1, 2007 or until a first fracture or death occurred. FN-BMD (g/cm2) at the RS1-3 (1997–1999) was measured by Dual-energy X-ray absorptiometry (DXA) using a Lunar DPX-L densitometer [23](Lunar Radiation Corp., Madison, WI, USA) and analyzed with DPX-IQ v.4.7d software whereas at the RS1-4 (2002–2004), FN-BMD was measured using a GE Lunar Prodigy bone densitometer. No cross-calibration between the two measures was performed. From the FN-BMD, sex-specific T-scores were calculated using the NHANES reference population [24]. Peak bone mass, as converted to the corresponding Lunar value, was 1.04 ± 0.14 g/cm2 for women and 1.13 ± 0.16 g/cm2 for men. Osteoporosis was defined as a T-score below -2.5 SD whereas osteopenia as a T-score between -1.0 and -2.5. Hip structural analysis [25] was used to measure HBG from the DXA scans of the femur narrow neck region as described previously [26].

### Assessment of Covariates

At the third visit, smoking habits were coded as current, and former/never. BMI was calculated as weight (in kg)/height (in m2). Information on medication use at the third round visit included the use of diuretics, hormonal replacement therapy, systemic corticosteroids, drugs for bone and other musculoskeletal diseases. A faller was defined as an individual with a history of one, two, or more falls without precipitating trauma (e.g., car accident or sport injury) in the 12 months preceding the interview at the third round visit. Falling frequency was then recorded as never or at least one. At the third visit to the research center, the total weekly duration of physical activity was assessed by an adapted version of the Zutphen Physical Activity Questionnaire and the LASA Physical Activity Questionnaire. The Dutch Healthy Diet (DHD)-index assessed at the first wave of the Rotterdam Study (1989–1993), was used to take into account overall dietary quality. The DHD represents compliance to the Dutch Guidelines for a Healthy Diet as assessed from the FFQ at baseline [27].

### Statistical Analysis

Continuous variables were reported as mean ± SD unless stated otherwise and categorical variables were presented as percentages. Linear regression models and logistic regression were used to determine the cross-sectional association between MS and the number of MS components with FN-BMD, HBG parameters and osteoporosis. For the longitudinal association of MS and its components with FN-BMD, linear regression models were fitted in generalized estimated equations (GEE) with exchangeable correlation structure adjusting for the within-subject correlations due to the repeated measurements of FN-BMD in the same individual (partial *Pearson* correlation = 0.93 and intra-class correlation = 0.96) [28]. Risk of incident fractures was evaluated for the association with MS and the number of different MS components using Cox proportional hazard regression models. The proportional hazard assumption of the Cox model was checked by the visual inspection of log minus log plots and by performing an interaction test with time. Models for fracture, FN-BMD, and hip geometry were corrected for potential confounders, including age, height, smoking status, physical activity, alcohol intake, fallings in the last 12 months, use of diuretics drugs, use of hormone replacement therapy, use of corticosteroids drugs, use of drugs for bone and other musculoskeletal diseases and Dutch Healthy Diet Index; cross-sectional FN-BMD and HBG were additionally adjusted for BMI and weight respectively, longitudinal analysis on FN-BMD were additionally adjusted for BMI and the two time points FN-BMD measurements were performed (index time) (to adjust also for the lack of cross-calibration between the two measures of BMD) and the interaction between MS (or MS component) and the index time whereas fracture analysis were further adjusted for weight. To analyze the relationship between individual features of MS and FN-BMD, GEE was performed after adjusting for the possible covariates described above with respect to the presence of each component. All analyses were conducted separately for men and women because of gender differences in MS and bone parameters (also the formal tests of interaction (sex×MS) in BMI adjusted models were statistically significant). To correct for multiple testing, a two-tailed *P* value of 0.025 or less was considered as statistically significant.

To examine if diabetic individuals could influence the associations, we re-ran all analyses excluding subjects with MS who had diabetes mellitus at the third round visit. To adjust for potential bias associated with missing data we used multiple imputation procedure (N = 5 imputations). Rubin’s method was used for the pooled regression coefficients (β) and 95% Confidence Intervals. All analyses were performed using SPSS statistical software (SPSS, version 21.0; SPSS Inc, Chicago, Illinois).

## Results

The baseline characteristics of the study population included in the analysis of MS with FN-BMD are shown in Table 1. Six hundred ninety eight (45.7%) female and 435 (37.3%) male participants had MS. On average, female participants were older than men (72.38 vs. 72.04) and were more likely to have osteoporosis (18.2% vs. 14.8%). Anthropometric, lifestyle and other characteristics of the excluded participants did not substantially differ from the participants included in the study (data not shown).

**Table 1:** Baseline characteristics for participants.

	Women (N = 1,527)	Men (N = 1,166)
Age	72.38 ±6.81	72.04 ±6,51
Metabolic Syndrome (n, %)	698 (45.7)	435 (37,3)
Bone mineral density, 1st round (g/cm^2^)	0.82 ± 0.14	0.93 ± 0.14
Bone mineral density, 2^nd^ round (g/cm^2^)*	0.82 ± 0.13	0.92 ± 0.13
Cortical thickness (cm)**	0.13 ± 0.04	0.15 ± 0.03
Bone width (cm)**	2.93 ± 0.32	3.37 ± 0.30
Section modulus (cm^3^)**	0.97 ± 0.31	1.41 ± 0.34
Cortical buckling ratio**	13.50 ± 5.00	13.15 ± 3.96
Osteoporosis (n, %)	278 (18.2)	172 (14.8)
Osteopenia (n, %)	855 (56.0)	640 (54.6)
Diabetes Mellitus (n,%)	194 (12.7)	172 (14.8)
BMI (kg/m^2^)	27.15 ±4.25	26.35 ±3.19
Smoking (Yes) (n,%)	216 (14.0)	201 (17.2)
Physical activity (min/week)	2820.62 ±1103.74	2519.15 ±1178
Alcohol intake (g/day)	1,57 (396821.4)	4.29 (79364.3)
Dutch Healthy Diet-Index	50.84 ±9.92	45.55 ±9.78
Fallings in the last 12 months (n, %)	415 (27.2)	225 (19.3)
Diuretic drugs (n,%)	264 (17.3)	163 (14.0)
HRT (n,%)	66 (4.3)	3 (0.26)
Corticosteroid drugs (n,%)	51 (3.3)	25 (2.1)
Bone drugs (n,%)	56 (3.86)	6 (0.53)
Other musculoskeletal drugs (n,%)	32 (2.1)	6 (0.53)

HRT: Hormone replacement therapy

*999 female and 768 male individuals with available measure of BMD at the second round

**1,403 female and 1,118 male individuals with available measure of hip bone geometry

### Cross-sectional association between Metabolic Syndrome, FN-BMD, Hip Bone Geometry and Osteoporosis

**Fig 1:**
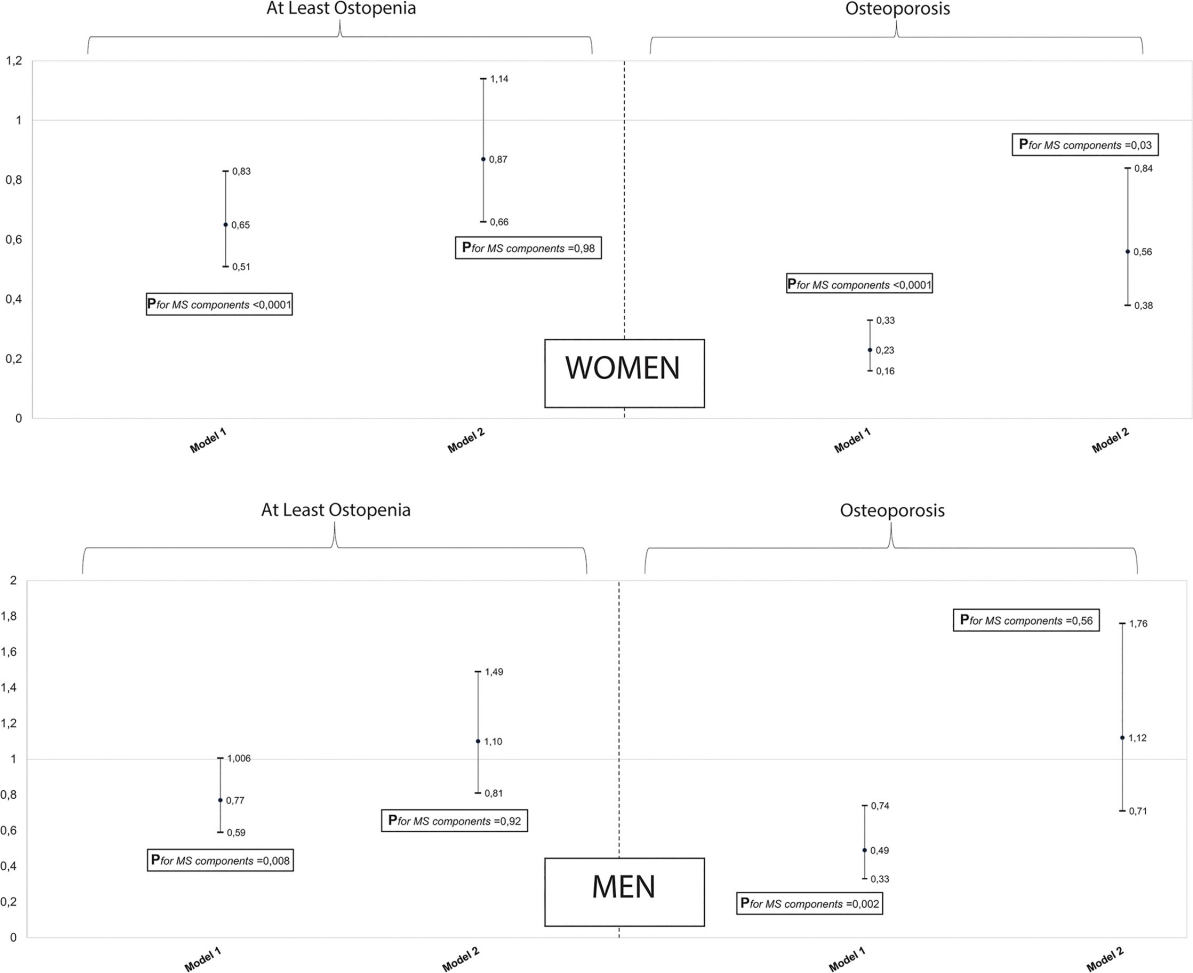
The association between metabolic syndrome, osteopenia and osteoporosis in women and men. Reference group are subjects with no osteopenia, neither osteoporosis: Confounders include age, body mass index, height, smoking status, physical activity, alcohol intake, fallings in the last 12 months, use of diuretics drugs, use of hormone replacement therapy, use of corticosteroids drugs, use of drugs for bone and other musculoskeletal diseases and Dutch Healthy Diet Index.

In females, in age-adjusted models, MS was associated with higher FN-BMD (β = 0.056, *P* = 4.5 10^−17^), higher cortical thickness (β = 0.01, *P* = 6.2 10^−07^), higher section modulus (β = 0.053, *P* = 0.001), lower cortical buckling ratio (β = -1.93, *P* = 2.2 10^−11^) whereas no association was found with bone width (β = -0.033, *P* = 0.055) (Table 2). After additional adjustments for confounding by BMI, height, lifestyle factors and medication use, the associations were attenuated or reversed (Table 2); however MS was still positively associated with FN-BMD (β = 0.017, *P* = 0.010), bone width (β = -0.054, *P* = 0.003) and cortical buckling ratio (β = -0.81, *P* = 0.003) (Table 2). Similarly, in the multivariable models, the number of MS components was positively associated with FN-BMD (β = 0.006,*P* = 0.012), femoral neck width (β = -0.026, *P*<0.001) and cortical buckling ratio (β = -0.26, *P* = 0.011) whereas no association was observed between MS and cortical thickness or section modulus in females (*P*>0.025) (Table 2). Also, MS and the number of its components, were inversely associated with osteopenia and osteoporosis in age-adjusted models (osteopenia: OR = 0.65, *P* = 3.2 10^−04^, P_*for no*. *of MS components*_<0.0001; osteoporosis: OR = 0.23, *P* = 3.3 10^−16^, P_*for no*. *of MS components*_<0.0001) (Fig 1). After adjustment for BMI although attenuated, the inverse association between MS and osteoporosis remained significant (OR = 0.56, *P* = 0.006) (Fig 1). Also, a positive association was observed between the number of MS features and osteoporosis in the multivariable model P_*for no*. *of MS components*_ = 0.03) (Fig 1). Additional adjustment for other confounders did not affect the results (data not shown). In males, MS was associated with higher FN-BMD (β = 0.031, *P* = 7.4 10^−05^), higher cortical thickness (β = 0.009, *P* = 9.3 10^−07^), higher section modulus (β = 0.096, *P* = 3.0 10^−07^) and lower cortical buckling ratio (β = -0.88, *P* = 2.2 10^−04^) in age-adjusted models (Table 2). However, after adjustment for BMI and height, MS was neither statistically significantly associated with FN-BMD nor with hip bone geometry parameters (Table 2). Similarly, there was no association between number of MS components, FN-BMD and hip bone geometry in the fully adjusted models (Table 2). Also, in age-adjusted models, MS was inversely associated with osteoporosis but not with osteopenia (osteopenia: OR = 0.77, *P* = 0.056, P_*for no*. *of MS components*_ = 0.008; osteoporosis: OR = 0.49, *P* = 0.001, P_*for no*. *of MS components*_ = 0.002) (Fig 1). However, no association was observed between MS and its components with osteopenia or osteoporosis after adjustment for BMI (Fig 1) and other confounders (data not shown).

**Table 2:** The cross-sectional association of metabolic syndrome with bone mineral density and bone geometry.

Women			Men		
**FN-BMD, n = 1,527**			**FN-BMD, n = 1,166**		
Metabolic Syndrome	β (95%CI)	P-value	Metabolic Syndrome	β (95%CI)	P-value
Model 1	0.056 (0.043; 0.069)	4.5 10^−17^	Model 1	0.031 (0.015; 0.047)	7.4 10^−5^
Model 2	0.018 (0.003; 0.030)	0.007	Model 2	-0.005 (-0.014; 0.003)	0.55
Model 3	0.017 (0.004; 0.030)	0.10	Model 3	-0.002 (-0.020; 0.015)	0.82
No. of MS components			No. of MS components		
Model 1	0.022 (0.017; 0.026)	8.08 10^−20^	Model 1	0.012 (0.006; 0.018)	9.6 10^−5^
Model 2	0.007 (0.002;0.011)	0.009	Model 2	-0.004 (-0.007; 0.001)	0.30
Model 3	0.006 (0.001; 0.011)	0.012	Model 3	-0.002 (-0.009; 0.005)	0.53
**Cortical thickness, n = 1,403**			**Cortical thickness, n = 1,118**		
Metabolic Syndrome	β (95%CI)	P-value	Metabolic Syndrome	β (95%CI)	P-value
Model 1	0.010 (0.005; 0.014)	6.2 10^−7^	Model 1	0.009 (0.005; 0.012)	9.3 10^−7^
Model 2	0.001 (-0.003; 0.005)	0.69	Model 2	-0.001 (-0.003; 0.001)	0.56
Model 3	0.001 (-0.004; 0.006)	0.73	Model 3	-0.0005 (-0.004; 0.003)	0.80
No. of MS components			No. of MS components		
Model 1	0.004 (0.003; 0.006)	1.33 10^−8^	Model 1	0.003 (0.002; 0.005)	7.3 10^−7^
Model 2	0.001 (-0.001; 0.002)	0.42	Model 2	-0.001 (-0.002; 0.000)	0.26
Model 3	0.001 (-0.001; 0.002)	0.49	Model 3	-0.001 (-0.002; 0.001)	0.45
**Bone width, n = 1,403**			**Bone width, n = 1,118**		
Metabolic Syndrome	β (95%CI)	P-value	Metabolic Syndrome	β (95%CI)	P-value
Model 1	-0.033 (-0.068; 0.001)	0.055	Model 1	0.006 (-0.003; 0.042)	0.76
Model 2	-0.051 (-0.09; -0.015)	0.005	Model 2	-0.030 (-0.068; 0.008)	0.12
Model 3	-0.054 (-0.091; -0.018)	0.003	Model 3	-0.029 (-0.068; 0.010)	0.14
No. of MS components			No. of MS components		
Model 1	-0.015 (-0.027; -0.002)	0.021	Model 1	0.001 (-0.014; 0.015)	0.93
Model 2	-0.025 (-0.038; -0.011)	0.0003	Model 2	-0.014 (-0.029; 0.001)	0.076
Model 3	-0.026 (-0.039; -0.012)	0.0002	Model 3	-0.013 (-0.029; 0.002)	0.097
**Section Modulus, n = 1,403**			**Section Modulus, n = 1,118**		
Metabolic Syndrome	β (95%CI)	P-value	Metabolic Syndrome	β (95%CI)	P-value
Model 1	0.053 (0.021; 0.086)	0.001	Model 1	0.096 (0.056; 0.136)	3.0 10^−7^
Model 2	-0.029 (-0.062; 0.003)	0.08	Model 2	-0.028 (-0.068; 0.011)	0.159
Model 3	-0.03 (-0.063; 0.003)	0.078	Model 3	-0.021 (-0.061; 0.019)	0.30
No. of MS components			No. of MS components		
Model 1	0.024 (0.012; 0.036)	5.6 10^−5^	Model 1	0.036 (0.021; 0.052)	5.0 10^−7^
Model 2	-0.011 (-0.023; 0.002)	0.09	Model 2	-0.017 (-0.032; -0.001)	0.036
Model 3	-0.011 (-0.023; 0.001)	0.08	Model 3	-0.014 (-0.030; 0.002)	0.094
**Cortical Buckling Ratio, n = 1,403**			**Cortical Buckling Ratio, n = 1,118**		
Metabolic Syndrome	β (95%CI)	P-value	Metabolic Syndrome	β (95%CI)	P-value
Model 1	-1.93 (-2.42; -1.43)	2.2 10^−11^	Model 1	-0.88 (-1.35; -0.41)	2.2 10^−4^
Model 2	-0.79 (-1.32; -0.26)	0.004	Model 2	0.036 (-0.23; 03037)	0.89
Model 3	-0.81 (-1.34; -0.27)	0.003	Model 3	-0.049 (-0.56; 0.46)	0.85
No. of MS components			No. of MS components		
Model 1	-0.71 (-0.89; -0.53)	3.1 10^−12^	Model 1	-0.314 (-0.50; -0.13)	0.001
Model 2	-0.25 (-0.45; -0.06)	0.012	Model 2	0.101 (-0.003; 0.21)	0.33
Model 3	-0.26 (-0.46; -0.06)	0.011	Model 3	0.07 (-0.14; 0.27)	0.51

MS, metabolic syndrome; FN-BMD, femoral neck bone mineral density

Model 1: Adjusted for age

Model 2: Model 1 +body mass index and height for FN-BMD and weight + height for hip bone geometry parameters.

Model 3: Model 2 + smoking status, physical activity, alcohol intake, fallings in the last 12 months, use of diuretics drugs, use of hormone replacement therapy, use of corticosteroids drugs, use of drugs for bone and other musculoskeletal diseases and Dutch Healthy Diet Index.

### Longitudinal Association between Metabolic Syndrome and FN-BMD

Similar to the cross-sectional analysis, MS and the number of its features was positively associated with FN-BMD (β = 0.028, *P* = 0.001; P_*for no*. *of MS components*_ = 0.001) in the multivariable model in females, which tended to go away across time (interaction MS x index time: β = -0.008, p = 0.031; interaction MS component x index time: β = -0.003, p = 0.021). No association was observed between MS or its features and adjusted FN-BMD in males (Table 3).

**Table 3:** The longitudinal association of metabolic syndrome with bone mineral density.

Women (N = 1,527)			Men (N = 1,166)#		
**Metabolic syndrome (Yes vs. No)**	**FN-BMD**	**P-value**	**Metabolic syndrome (Yes vs. No)**	**FN-BMD**	**P-value**
Model 1: β, 95% CI	0.063 (0.048; 0.079)	2.44 10^−15^	Model 1: β, 95% CI	0.031 (0.015; 0.047)	0.0001
Model 2: β, 95% CI	0.028 (0.015; 0.042)	0.001	Model 2: β, 95% CI	-0.006 (-0.016; 0.004)	0.58
Model 3: β, 95% CI	0.028 (0.012; 0.043)*	0.001	Model 3: β, 95% CI	-0.002 (-0.022; 0.017)	0.83
**No. of MS components (continuous)**			**No. of MS components (continuous)**		
Model 1: β, 95% CI	0.025 (0.019; 0.030)	5.4 10^−20^	Model 1: β, 95% CI	0.013 (0.006; 0.020)	0.0003
Model 2: β, 95% CI	0.011 (0.005;0.016)	0.0004	Model 2: β, 95% CI	-0.003 (-0.007; 0.001)	0.46
Model 3: β, 95% CI	0.010 (0.004; 0.016)**	0.001	Model 3: β, 95% CI	-0.001 (-0.009; 0.006)	0.75

MS, metabolic syndrome; FN-BMD, femoral neck bone mineral density

Model 1: Adjusted for age and type of DXA scan

Model 2: Model 1 +body mass index and height

Model 3: Model 2 + smoking status, physical activity, alcohol intake, fallings in the last 12 months, use of diuretics drugs, use of hormone replacement therapy, use of corticosteroids drugs, use of drugs for bone and other musculoskeletal diseases and Dutch Healthy Diet Index.

*index time (time points when the DXA measurements were performed), β = -0.012, p<0.001; interaction MS x index time: β = -0.008, p = 0.031

** index time, β = -0.012, p<0.001; interaction MS component x index time: β = -0.003, p = 0.021

#no significant interaction between MS (or MS component) and index time (p>0.50) was observed in any of the analysis in men and therefore data are not shown

### Association between Metabolic Syndrome and Fracture Risk

In females, no association was observed between MS and any type of fractures, neither in age and gender adjusted model, nor in the multivariable model (HR = 0.91: 95%CI: 0.73–1.15) (Table 4). Also no association was observed for non-vertebral fractures (HR = 0.94: 95%CI: 0.73–1.21) or vertebral fractures (HR = 0.83: 95%CI: 0.56–1.24) (Table 3). Similarly, there was no significant association between MS with any type of fractures or with subtypes of fractures in males (Table 4). Nevertheless, in males, significant inverse associations were observed between numbers of MS components with any type of fractures (*P* = 0.015) and with non-vertebral fractures (*P* = 0.017) (Table 3).

**Table 4:** Metabolic syndrome and fracture risk.

Women			Men		
All Fractures (371)			All Fractures (147)		
Metabolic Syndrome	Hazard ratio (95%CI)	P-value	Metabolic Syndrome	Hazard ratio (95%CI)	P-value
Model 1	0.85 (0.69–1.04)	0.12	Model 1	0.85 (0.60–1.20)	0.36
Model 2	0.91 (0.72–1.14)	0.40	Model 2	0.74 (0.69–1.08)	0.12
Model 3	0.91 (0.73–1.15)	0.43	Model 3	0.68 (0.46–1.006)	0.054
No. of MS components			No. of MS components		
Model 1	0.94 (0.87–1.02)	0.11	Model 1	0.92 (0.81–1.06)	0.24
Model 2	0.97 (0.89–1.05)	0.44	Model 2	0.86 (0.74–1.001)	0.055
Model 3	0.97 (0.89–1.06)	0.47	Model 3	0.82 (0.70–0.9964)	0.015
Non-Vertebral Fractures (307)			Non-Vertebral Fractures (102)		
Metabolic Syndrome	Hazard ratio (95%CI)	P-value	Metabolic Syndrome	Hazard ratio (95%CI)	P-value
Model 1	0.90 (0.72–1.13)	0.36	Model 1	0.89 (0.59–1.35)	0.59
Model 2	0.94 (0.73–1.21)	0.63	Model 2	0.69 (0.55–1.88)	0.12
Model 3	0.94 (0.73–1.21)	0.61	Model 3	0.64 (0.40–1.03)	0.068
No. of MS components			No. of MS components		
Model 1	0.96 (0.89–1.05)	0.38	Model 1	0.94 (0.79–1.10)	0.42
Model 2	0.98 (0.89–1.08)	0.70	Model 2	0.83 (0.68–1.01)	0.047
Model 3	0.98 (0.89–1.08)	0.66	Model 3	0.75 (0.66–0.96)	0.017
Vertebral Fractures (123)			Vertebral Fractures (62)		
Metabolic Syndrome	Hazard ratio (95%CI)	P-value	Metabolic Syndrome	Hazard ratio (95%CI)	P-value
Model 1	0.68 (0.47–0.98)	0.039	Model 1	0.65 (0.37–1.13)	0.13
Model 2	0.80 (0.53–1.19)	0.27	Model 2	0.67 (0.49–1.25)	0.20
Model 3	0.83 (0.56–1.24)	0.36	Model 3	0.60 (0.32–1.14)	0.12
No. of MS components			No. of MS components		
Model 1	0.84 (0.73–0.96)	0.01	Model 1	0.84 (0.6–1.03)	0.10
Model 2	0.86 (0.76–1.03)	0.11	Model 2	0.85 (0.75–1.07)	0.17
Model 3	0.90 (0.77–1.05)	0.18	Model 3	0.81 (0.64–1.03)	0.09

“( )”, number of fractures

Model 1: Adjusted for age

Model 2: Model 1 +Height and Weight

Model 3: Model 2 + smoking status, physical activity, alcohol intake, fallings in the last 12 months, use of diuretics drugs, use of hormone replacement therapy, use of corticosteroids drugs, use of drugs for bone and other musculoskeletal diseases and Dutch Healthy Diet Index.

### Additional Analysis

S1 Table shows the effect of individual components of MS on the adjusted FN-BMD. In both genders, elevated glucose levels were associated with higher BMD (in females: β = 0.016, *P* = 0.01; in males: β = 0.022, *P* = 0.004). In women, but not in men, HDL-cholesterol was positively associated with FN-BMD (β = 0.013, P = 0.01). In men but not in women, waist circumference component was inversely associated with FN-BMD (in males: β = -0.030, *P* = 0.004). No association was observed between other components of MS and adjusted FN-BMD in either gender.

Exclusion of subjects with MS having diabetes mellitus (194 females and 172 males) did not substantially change any of the results (data not shown). Moreover, substitution in the multivariable models of BMI with weight and vice versa, did not change any of the results (data not shown).

## Discussion

To our knowledge, this is the first study to simultaneously examine the association between metabolic syndrome, FN-BMD, HBG parameters, osteoporosis and fracture risk by using the most recent definition of MS. Females with MS, have significantly higher BMD, narrower bone at the hip, and increased bone instability (lower buckling ratio), lower odds of having osteoporosis than non-MS individuals, independent of BMI. In males, the positive association of MS with BMD and HBG geometry was explained by BMI. Our results on fracture risk, suggest no consistent influence of MS in either gender.

According to the observational data in the past decade, the relationship between MS and its components, BMD and fracture risk is controversial. In line with our findings, a recent meta-analysis showed that MS may have beneficial influence on BMD in Caucasian individuals [16]. Also, another meta-analysis on MS and fracture risk, concluded that individuals with MS are not at higher risk of having fractures [17]. MS is a cluster of conditions, interacting with each other, and therefore the mechanism behind the effect of MS on BMD and fractures risk is complicated and has not yet been investigated in detail. Although, the association between individual components of MS and bone metabolism have been extensively studied, the results are yet inconclusive. (1) For example, central obesity has been associated with higher BMD in some studies, but some others and our study as well have shown detrimental effects[29,30]. Also, obesity, is a risk factor for fractures of the humerus and ankle, but protects against fractures of hip and vertebral bodies [31–33]. (2) Elevated glucose levels have been linked to better, worse or similar bone outcomes [18,34,35]. (3) Hypertension is postulated to be associated with low bone mass due to urinary calcium excretion and therefore increase fracture risk [36], but, contrary to common belief, intensive antihypertensive treatment was not associated with an increased risk of falls or non-spine fractures in patients with type 2 diabetes in the Action to Control Cardiovascular Risk in Diabetes (ACCORD) randomized trial [37]. Hypertriglyceridemia contributes to a lower risk of fractures, which may partly be explained by the interaction with protein matrix and bone minerals [12,38]. However, Kim et al [39] and Adami et al [13] found that high triglyceride levels and low HDL-cholesterol were negatively associated with BMD. Therefore, as observed in the current study, the combined effects of these components on bone metabolism may be beneficial or insignificant.

**Fig 2:**
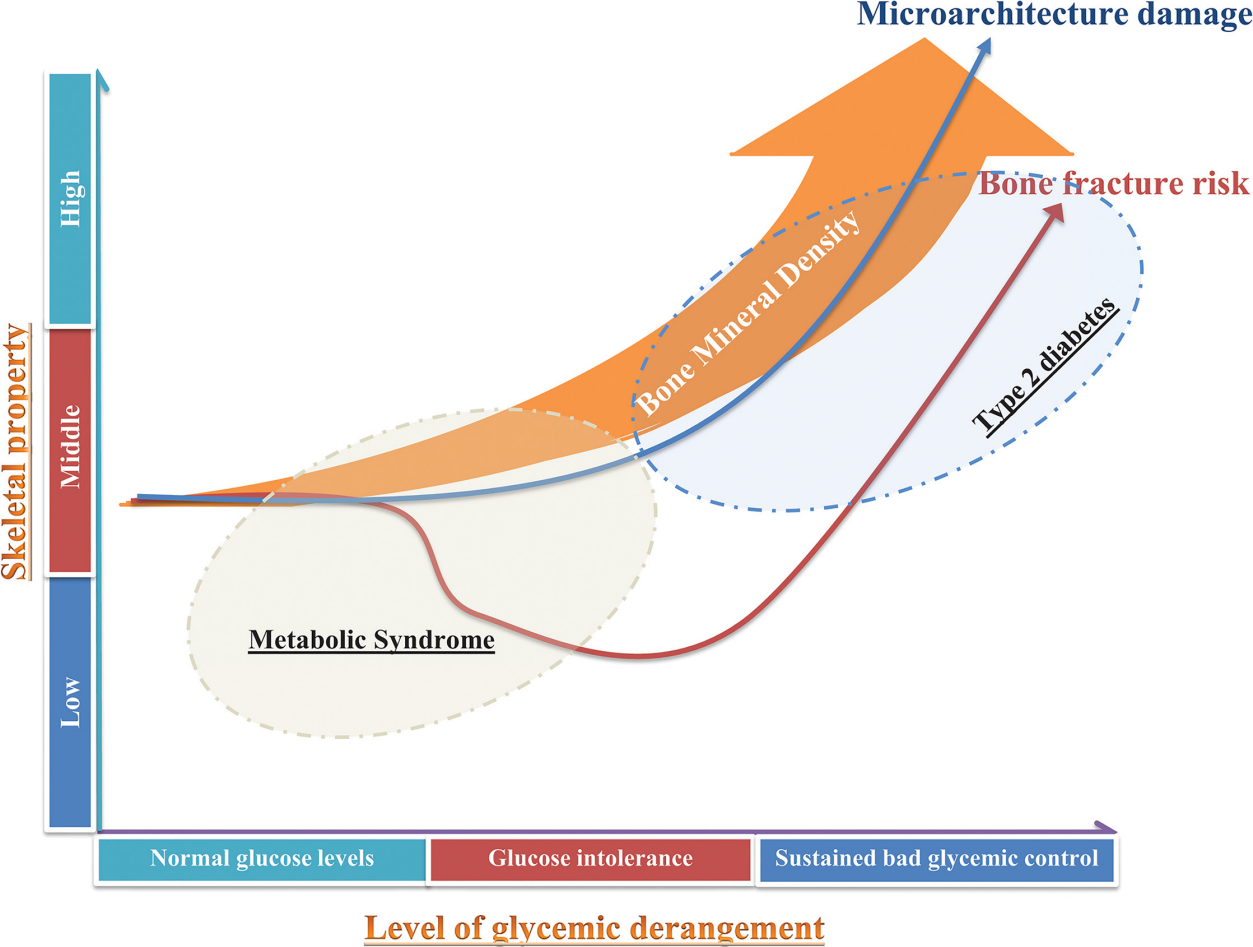
Level of glycemic derangement, bone architecture and fracture risk. Cartoon depicting the differences in bone mineral density, fracture risk and changes in bone microarchitecture across the stages of glucose derangement. Metabolic syndrome and diabetes mellitus individuals have higher BMD but do not experience yet an increase in fracture risk. With sustained bad glycemic control, the damage of bone microarchitecture represented by accumulation of microcracks and cortical porosity becomes a possibility which may explain the bone fragility and fracture susceptibility despite the observed increase in BMD. Drawing is not to scale.

The positive association between MS and BMD observed in the present study was mainly driven by glucose levels. MS is a risk factor for type 2 diabetes mellitus which has been associated with an increase in fracture risk. In contrast to MS individuals, evaluation of BMD may not be adequate for predicting fracture risk in patients with type 2 diabetes, who are unlikely to be diagnosed with osteoporosis and increased risk of fracture [10]. In the large Rotterdam Study, we previously showed that, similar to MS individuals, diabetic patients have higher BMD and stronger bone geometry, which would protect them against fractures [10]. However, in contrast to MS individuals, diabetic individuals have an increased fracture risk, which was shown to be driven by poor glycemic control [10]. We postulate that increased bone fragility may be caused by chronically elevated glucose levels, which may lead to accumulation of microcracks and/or cortical porosity. MS is a health condition which predisposes to type 2 diabetes mellitus, but may not yet be characterized by chronically elevated glucose levels. In the present study, the positive association between MS and BMD persisted also after exclusion of subjects with MS who had diabetes mellitus. Thus, it is likely that, microcracks and/or cortical porosity may not be present in MS individuals which may explain why MS subjects do not yet experience a high risk of fractures (Fig 2). Follow-up studies focusing on the health life trend of MS individuals in relation to bone are thus needed.

The current study shows that no gender differences are observed between MS and unadjusted BMD and fracture risk, but that gender differences become prominent after adjustment for BMI and that in men, the observed positive association between MS and BMD and hip bone geometry parameters is explained by obesity. Similar to our observations, the meta-analysis investigating the association between MS and unadjusted BMD concluded that there were no gender differences [16]. Also, the recent meta-analysis examining the association between MS and fracture risk did not observed any difference between men and women with similar estimates to our study [17]. In contrast, gender differences in the association between MS and BMI-adjusted BMD have been previously described, showing less beneficial effects of MS on bone health in men than in women [39,40]. In contrast to our results, both studies observed a decrease in FN-BMD with increasing number of MS components in men, but not in women. However, both studies were characterized by other ethnicities or were performed in a younger population. It has been shown that the association between MS and BMD differs by ethnicity [16]. Also, BMD declines by age and this decline differs by gender [41]. In a twin study, fat mass and body fat distribution seem to have different relationships with BMD according to gender and age [42]. Since fat deposition differs according to gender, it has been hypothesized that mechanical effect and estrogen synthesis predominate in women, while bone-deleterious effects of fat, related to oxidative stress and chronic inflammation, predominate in men [43]. Therefore, bone mass in males with MS may be more influenced by visceral fat than in women which may explain the dilution of the association after adjustment for BMI and the relationship between waist circumference and BMD in men and not in women.

There are several strength of our present study. First, this is a large prospective population based study of 3,458 individuals, with comprehensive follow up of more than 6.7 years on average. We were also able to examine both cross-sectional and longitudinal the associations between MS, BMD and osteoporosis. Additionally, the present study had various indices of bone outcomes available, including hip bone geometry parameters and fracture incidence. Moreover, in contrast to other studies, we used the most recent definition of MS. Yet our study has some limitations. We did not have measures of glucose control, such as HBA1C which could have strengthened our results. Secondly, the subjects in this study were only caucasians. Ethnic differences in the association between MS and/or its components and bone have been previously reported. Thus, our findings may not be extended to non-caucasian groups.

In conclusion, MS is associated with higher BMD, increase instability and narrower bone, which is mainly driven by elevated glucose levels. In men but not in women, higher FN-BMD was mainly explained by body mass index and body fat distribution. We postulate that the bone of MS individuals is not yet characterized by an accumulation of microcracks (cortical porosity) that would reflect sustained impairment of bone structure, and therefore yet an increased risk of fragility, as observed in diabetic individuals. These highlight the importance of maintaining glycemic control in individuals with MS to pervert skeletal complications and preserve bone health.

## Supporting Information

S1 Fig Flow chart of female study participants. (PDF)

S2 Fig Flow chart of male study participants. (PDF)

S1 Table The association of individually components of metabolic syndrome with bone mineral density*. FN-BMD: femoral neck bone mineral density. *Waist circumference: ≥102 cm for men or ≥88 cm for women; Triglyceride: ≥150 mg/Dl; HDL-cholesterol:≤40 mg/dL for men or ≤50 mg/dL for women; fasting glucose ≥100 mg/Dl; blood pressure: systolic BP ≥130 and/or diastolic BP ≤85 mmHg. ^1^: triglyceride component, HDL-cholesterol component, hypertension component, glucose component, age, index time, BMI and height. smoking status, physical activity, alcohol intake, fallings in the last 12 months, use of diuretics drugs, use of hormone replacement therapy, use of corticosteroids drugs, use of drugs for bone and other musculoskeletal diseases and Dutch Healthy Diet Index. ^2^: waist circumference component, HDL-cholesterol component, hypertension component, glucose component, age, index time, BMI and height. smoking status, physical activity, alcohol intake, fallings in the last 12 months, use of diuretics drugs, use of hormone replacement therapy, use of corticosteroids drugs, use of drugs for bone and other musculoskeletal diseases and Dutch Healthy Diet Index. ^3^: waist circumference component, triglyceride component, hypertension component, glucose component, age, index time, BMI and height. smoking status, physical activity, alcohol intake, fallings in the last 12 months, use of diuretics drugs, use of hormone replacement therapy, use of corticosteroids drugs, use of drugs for bone and other musculoskeletal diseases and Dutch Healthy Diet Index. ^4^: waist circumference component, triglyceride component, HDL-cholesterol component, glucose component, age, index time, BMI and height. smoking status, physical activity, alcohol intake, fallings in the last 12 months, use of diuretics drugs, use of hormone replacement therapy, use of corticosteroids drugs, use of drugs for bone and other musculoskeletal diseases and Dutch Healthy Diet Index. ^5^: waist circumference component, triglyceride component, HDL-cholesterol component, hypertension component, age, index time, BMI and height. smoking status, physical activity, alcohol intake, fallings in the last 12 months, use of diuretics drugs, use of hormone replacement therapy, use of corticosteroids drugs, use of drugs for bone and other musculoskeletal diseases and Dutch Healthy Diet Index.
(DOCX)
