# Supplementary figures and images for: The Association between Metabolic Syndrome, Bone Mineral Density, Hip Bone Geometry and Fracture Risk: The Rotterdam Study
Source: PLoS One. 2015 Jun 12;10(6):e0129116. doi: 10.1371/journal.pone.0129116 (PMC4466576; doi:10.1371/journal.pone.0129116)

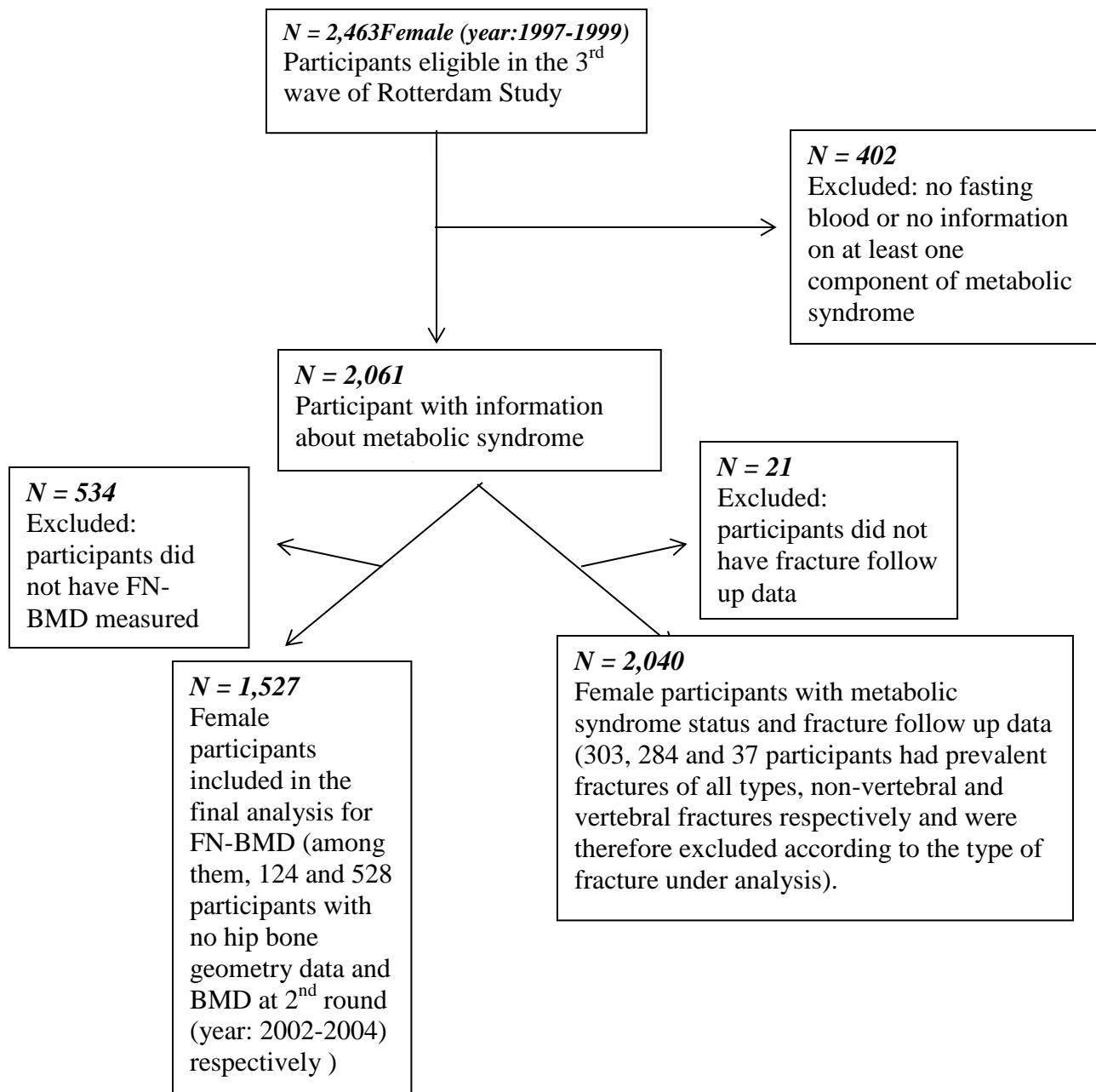

Supplement: S1 Fig — (PDF) [file pone.0129116.s001.pdf]

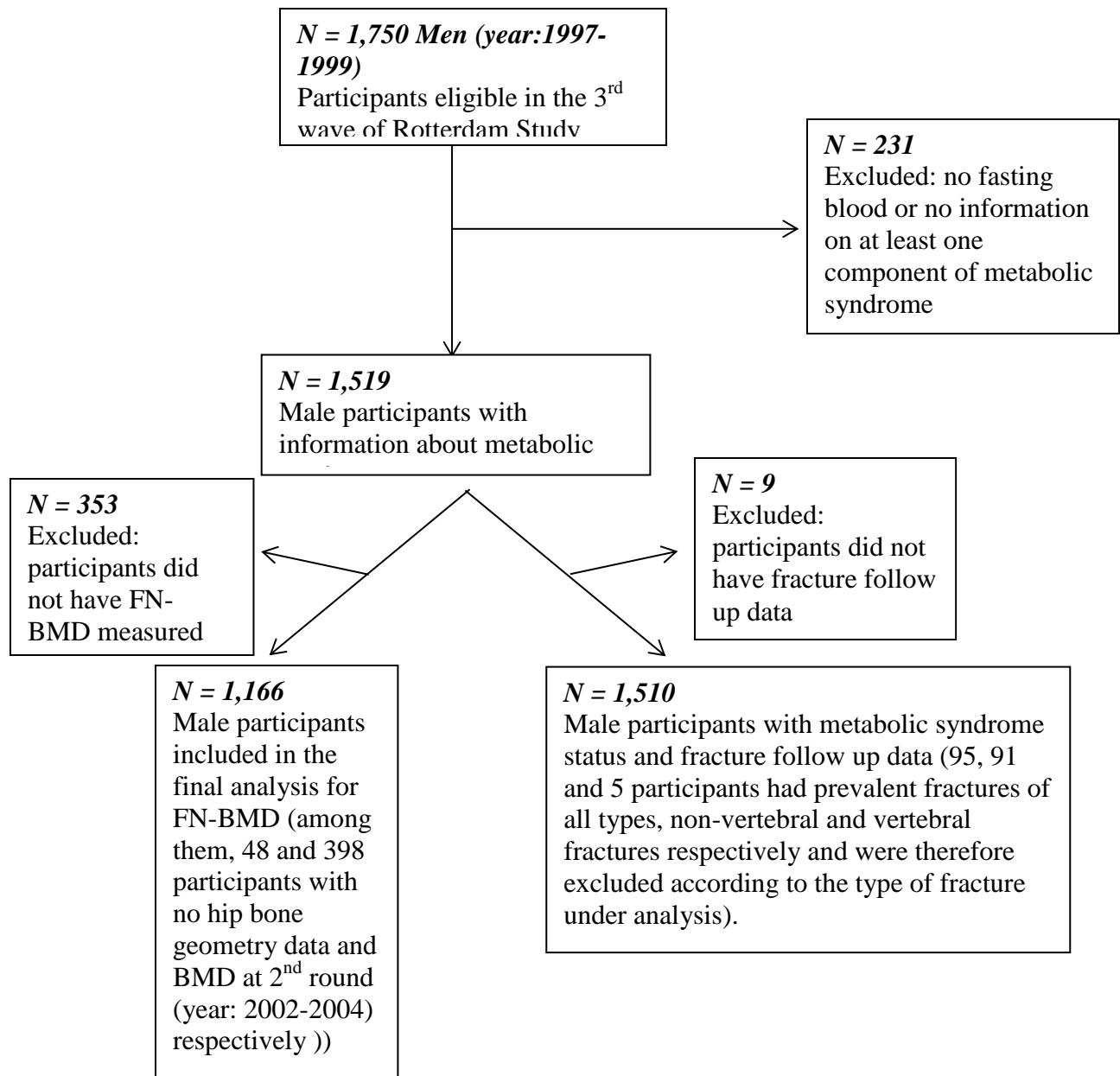

Supplement: S2 Fig — (PDF) [file pone.0129116.s002.pdf]
