# Supplementary material for: The Association between Metabolic Syndrome, Bone Mineral Density, Hip Bone Geometry and Fracture Risk: The Rotterdam Study
Source: PLoS One. 2015 Jun 12;10(6):e0129116. doi: 10.1371/journal.pone.0129116 (PMC4466576; doi:10.1371/journal.pone.0129116)
Supplement: S1 Table — FN-BMD: femoral neck bone mineral density. *Waist circumference: ≥102 cm for men or ≥88 cm for women; Triglyceride: ≥150 mg/Dl; HDL-cholesterol:≤40 mg/dL for men or ≤50 mg/dL for women; fasting glucose ≥100 mg/Dl; blood pressure: systolic BP ≥130 and/or diastolic BP ≤85 mmHg. 1: triglyceride component, HDL-cholesterol component, hypertension component, glucose component, age, index time, BMI and height. smoking status, physical activity, alcohol intake, fallings in the last 12 months, use of diuretics drugs, use of hormone replacement therapy, use of corticosteroids drugs, use of drugs for bone and other musculoskeletal diseases and Dutch Healthy Diet Index. 2: waist circumference component, HDL-cholesterol component, hypertension component, glucose component, age, index time, BMI and height. smoking status, physical activity, alcohol intake, fallings in the last 12 months, use of diuretics drugs, use of hormone replacement therapy, use of corticosteroids drugs, use of drugs for bone and other musculoskeletal diseases and Dutch Healthy Diet Index. 3: waist circumference component, triglyceride component, hypertension component, glucose component, age, index time, BMI and height. smoking status, physical activity, alcohol intake, fallings in the last 12 months, use of diuretics drugs, use of hormone replacement therapy, use of corticosteroids drugs, use of drugs for bone and other musculoskeletal diseases and Dutch Healthy Diet Index. 4: waist circumference component, triglyceride component, HDL-cholesterol component, glucose component, age, index time, BMI and height. smoking status, physical activity, alcohol intake, fallings in the last 12 months, use of diuretics drugs, use of hormone replacement therapy, use of corticosteroids drugs, use of drugs for bone and other musculoskeletal diseases and Dutch Healthy Diet Index. 5: waist circumference component, triglyceride component, HDL-cholesterol component, hypertension component, age, index time, BMI and height. smo [file pone.0129116.s003.docx]

| **Women (n=1,527)** | | | **Men (N=1,166)** | | |
| --- | --- | --- | --- | --- | --- |
| **Metabolic syndrome component (Yes vs. No)** | **FN-BMD** | **P-value** | **Metabolic syndrome component (Yes vs. No)** | **FN-BMD** | **P-value** |
| Waist Circumference: β, 95% CI^1^ | -0.013 (-0.029; 0.002) | 0.098 | Waist Circumference: β, 95% CI^1^ | **-0.030 (-0.05; -0.01)** | **0.004** |
| Triglyceride: β , 95% CI^2^ | 0.005 (-0.009; 0.019) | 0.47 | Triglyceride: β , 95% CI^2^ | 0.004 (-0.01; 0.02) | 0.66 |
| HDL-cholesterol: β , 95% CI^3^ | 0.013 (0.001; 0.027) | **0.01** | HDL-cholesterol: β , 95% CI^3^ | -0.010 (-0.028; 0.008) | 0.29 |
| Glucose :β , 95% CI^4^ | **0.016 (0.004; 0.028)** | **0.01** | Glucose :β , 95% CI^4^ | **0.022 (0.007; 0.0037)** | **0.004** |
| Blood pressure: β , 95% CI^5^ | 0.003 (-0.010; 0.016) | 0.64 | HTA: β , 95% CI^5^ | -0.014 (-0.032; 0.004) | 0.13 |
